# Metabolomics of Pigmented Rice Coproducts Applying Conventional or Deep Eutectic Extraction Solvents Reveal a Potential Antioxidant Source for Human Nutrition

**DOI:** 10.3390/metabo11020110

**Published:** 2021-02-15

**Authors:** Millena Cristina Barros Santos, Nathalie Barouh, Erwann Durand, Bruno Baréa, Mélina Robert, Valérie Micard, Valérie Lullien-Pellerin, Pierre Villeneuve, Luiz Claudio Cameron, Elizabeth P. Ryan, Mariana Simões Larraz Ferreira, Claire Bourlieu-Lacanal

**Affiliations:** 1 LabBio, Laboratory of Bioactives, Food and Nutrition Graduate Program, PPGAN, Federal University of State of Rio de Janeiro, UNIRIO, Rio de Janeiro 22290-240, Brazil; barrosmillena@gmail.com; 2 IMasS-LBP, Center of Innovation in MS-Laboratory of Protein Biochemistry, UNIRIO, Rio de Janeiro 22290-240, Brazil; cameron@unirio.br; 3 CIRAD, UMR IATE, 34398 Montpellier, France; nathalie.barouh@cirad.fr (N.B.); erwann.Durand@cirad.fr (E.D.); bruno.barea@cirad.fr (B.B.); melina.robert@cirad.fr (M.R.); pierre.villeneuve@cirad.fr (P.V.); 4 IATE, Univ Montpellier, INRAE, Institut Agro, 34000 Montpellier, France; Valerie.micard@supagro.fr (V.M.); valerie.lullien-pellerin@inrae.fr (V.L.-P.); 5 Department of Environmental and Radiological Health Sciences, Colorado State University, Fort Collins, CO 80523, USA; E.P.Ryan@colostate.edu

**Keywords:** phenolic compounds, pigmented rice bran, green solvent, metabolomics, UPLC-MS^E^, antioxidant

## Abstract

Rice bran (RB) corresponds to the outer layers of whole grain rice and contains several phenolic compounds (PCs) that make it an interesting functional food ingredient. PC richness is enhanced in pigmented RB varieties and requires effective ways of extraction of these compounds. Therefore, we investigated conventional and deep eutectic solvents (DES) extraction methods to recover a wide array of PCs from red and black RB. The RB were extracted with ethanol/water (60:40, *v/v*) and two DES (choline chloride/1.2-propanediol/water, 1:1:1 and choline chloride/lactic acid, 1:10, mole ratios), based on Generally Recognized as Safe (GRAS) components. Besides the quantification of the most typical phenolic acids of cereals, nontargeted metabolomic approaches were applied to PCs profiling in the extracts. Globally, metabolomics revealed 89 PCs belonging to flavonoids (52%), phenolic acids (33%), other polyphenols (8%), lignans (6%) and stilbenes (1%) classes. All extracts, whatever the solvents, were highly concentrated in the main phenolic acids found in cereals (37–66 mg/100 g in black RB extracts vs. 6–20 mg/100 g in red RB extracts). However, the PC profile was highly dependent on the extraction solvent and specific PCs were extracted using the acidic DES. The PC-enriched DES extracts demonstrated interesting DPPH scavenging activity, which makes them candidates for novel antioxidant formulations.

## 1. Introduction

Health-promoting properties of rice bran (RB) (*Oryza sativa* L.) have supported its application in food products for human consumption over the last decade. RB has been tested in the formulation of functional foods for human studies involving children and adults [1,2]. Indeed, RB nutritional profile is well diversified in essential and nonessential nutrients, and contains lysine-rich proteins [3], lipids [4], fibers and phytochemicals [5]. Additionally, RB consumption can positively modulate intestinal microbiota, contribute to the production of novel primary and secondary metabolites, to the regulation of intestinal immunity for the protection against bacterial infection with Salmonella [6,7] and to colon cancer prevention [8].

RB bioactivity is enhanced in pigmented rice varieties [9], very likely because of the higher levels and diversity of phenolic compounds (PCs). These pigmented rice gather usual phenolic acids found in nonpigmented varieties such as *p*-coumaric, syringic, vanillic, caffeic, sinapic, *p*-hydroxybenzoic, isoferulic and protocatechuic acids [9,10,11,12], but owe their color to flavonoids. For instance, anthocyanins such as cyanidin-3-*O*-glucoside and peonidin-3-*O*-glucoside and proanthocyanidins have already been identified in pigmented RB [13,14,15]. These pigmented-rice flavonoids and phenolic acids play an essential role against oxidative stress and inflammation. Such protective role was demonstrated in mouse [16,17] or human cell assays [18]. This antioxidant activity is reported to be even enhanced in black rice grains [10]. Among the wide range of bioactivity for pigmented RB potentially linked to its high PCs load, antiaging properties have been evidenced by Sunthonkun, et al. [19]. These authors have shown increased viability of yeast *Saccharomyces cerevisiae* when exposed to medium enriched in pigmented RB extracts. In addition, Khammanit, et al. [20] provided evidence for the antiproliferative properties of pigmented RB on HEK-293 cells. These antiproliferative effects were mediated by a reduction in ROS production, as well as an enhancement of antioxidant enzymes production.

Considering the high bioactivity of pigmented RB, it is an important and relevant scientific challenge to better examine their complex chemical PCs composition. Due to the wide range of PCs’ polarity, effective extraction is a key issue to properly elucidate the components of the pigmented RB extracts. This is generally handled by combining mixtures of organic solvents (e.g., methanol, isopropanol, chloroform, acetone) with water, but their efficiency is sometimes limited due to the low diversity of compounds extracted [21]. In addition, physically-assisted solvent extraction techniques have also been proposed to increase bioactive compounds extraction efficiency [22].

In this context, the application of a novel class of green solvents, called deep eutectic solvents (DES), has been proposed as a promising strategy to improve the extraction efficiency of bioactive compounds from coproducts [23]. DES show similar physical properties to the well-known ionic liquids (e.g., low vapor pressure, chemical and thermal stability, no flammability, high conductivity, high solubilizing capacity and low volatility) but with lower toxicity and better beneficial cost, accessibility and sustainability. What makes DES interesting is their unpredictable and strong solubilization capacity that was intensively used to provide enriched extracts with high phytochemical concentration and/or specificity [23]. For instance, Huang, et al. [24] successfully extracted the low soluble rutin reaching 95% recovery from buckwheat hull. In addition, DES can stabilize and protect molecules from degradation, mostly due to the supramolecular network of tightly interconnected molecules [25,26]. Depending on the DES composition, they may offer all characteristics to design atom economy, efficient, low cost and sustainable development for ready-to-use formulation, fully compatible with food and feed applications [23].

To resolve the complexity of DES extracts, “foodomics” can be applied since it represents a high-throughput approach able to elucidate the food chemical complexity by using chromatography as a separation method coupled with high-resolution mass spectrometry. Foodomics ensures simultaneously the coverage of diverse chemical specimens (*e.g.,* amino acids, lipids, carbohydrates, phytochemicals) [27,28]. These tools are used to identify and quantify chemical species in their ionized forms by measuring their mass/charge (*m/z*). In this study, we have investigated the potential of DES to extract PCs from pigmented RB in comparison to conventional ethanol/water solvent.

The chemometrics tools were applied for the chemical data generate characterization of the obtained extracts. Typical phenolic acids of cereals were also quantified with a high-performance liquid chromatography (HPLC) fitted with a diode-array detector (DAD).

The DPPH (2,2-diphenyl-1-picrylhydrazyl) test is based on the antioxidant activity of a hydrogen donor that will allow the reduction of DPPH [29]. Indeed, antioxidant activity is defined as the ability of an organism to protect itself against free radicals. This assay was performed to assess the capacity of extracts to stabilize radicals.

## 2. Results and Discussion

### 2.1. UPLC-MS^E^ Analyses to Unveil the Chemical Complexity and Diversity of PCs in Rice Bran Extracts

Considering the molecular complexity found in RB extracts, a putative identification of PCs and comparison between conventional and DES extracts with advanced UPLC-MS-MS tools was performed. Globally, a total of 89 PCs were tentatively identified in both extracts, including all extraction conditions. The putative PCs were identified following the recommendations of level 1 and 2 according to Sumner, et al. [30,31] considering mass to charge (*m/z*), retention time, isotopic similarity, precursor mass error, as well as the score and the fragmentation score for each identification attempt. These putative compounds are listed in the order of their retention time in Table 1. When all the parameters for the identification were equal, it was not possible to distinguish the compound and a multiple identification was proposed. In addition, compounds with the same m/z but with different retention times, were identified as isomers and were listed in the order of their retention time. In this work, 35 isomers of PCs were identified and 20 multiple identifications occurred.

**Figure 1:**
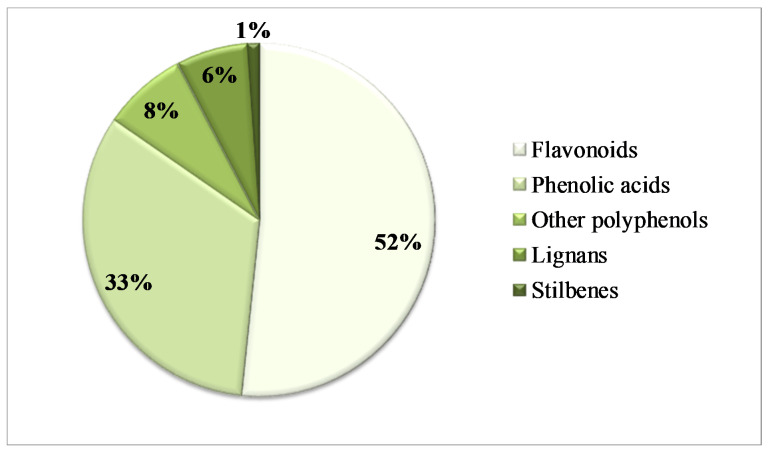
Percentage of number of tentatively identifications by class of phenolic compounds in all types of extracts whatever the RB.

The PCs identified in this study belonged to different chemical classes that were listed by decreasing number of occurrences in the extracts (Figure 1). Regardless of the RB types and conditions of extraction, the flavonoids were the most representative class of PCs with 52% of occurrences, followed by the phenolic acids (33%), other polyphenols (8%), lignans (6%) and stilbenes (1%).

As expected for pigmented rice, the flavonoids were the most abundant compounds present in the extracts, however only one anthocyanin (cyanidin 3-O-beta-D-sambubioside) was unambiguously identified. Considering the influence of the type of RB, a higher number of PCs was identified in the black rice RB (88) than in the red rice RB (81), which is in agreement with the literature [11]. Our results were in line with Pereira-Caro, et al. [32] who applied HPLC-PDA-MS^2^ to identify and quantify compounds in pigmented rice Camargue grains (black rice cultivar Artemide and red rice cultivar Tam Tam). They registered the presence of 34 PCs (34 present in black rice vs. 20 in red rice). In addition, these authors also pointed out that black rice was 14 times richer in PCs than red rice. Among the identified PCs, the most abundant class were flavonoids (subclasses: anthocyanins, flavones, flavonols, and flavan-3-ols) corresponding to 60% of the total. More recently, a metabolomic study with 17 RB cultivars originated from 11 countries identified 23 PCs [5]. Among them, seven were also identified in the present study and are highlighted in Table 1. The presence of chlorogenic acids corroborates the results found by Pang, Ahmed, Xu, Beta, Zhu, Shao and Bao [10] who identified two isomers of feruloylquinic acids. However, they did not detect the presence of caffeoylquinic acid and coumaroylquinic acid. In the present work, we could identify the presence of two isomers of feruloylquinic acids (*m/z* 367.1023 [M–H]^−^) and two isomers of coumaroylquinic acid (*m/z* 337.0916 [M–H]^−^) among the discriminant PCs. In addition, one isomer of caffeoylquinic acid (*m/z* 353.0863 [M–H]^−^) and one isomer of dicaffeoylquinic acid (*m/z* 515.1221 [M–H]^−^) were also identified (Table 1).

Although the presence of various anthocyanins has not been evidenced, PCs closely related to anthocyanins metabolism [33] have been identified such as dihydroquercetin, quercetin (the fourth most abundant PC identified in this study) and myricetin.

### 2.2. Focus on the Tentative Identification of Phenolic Compounds by Solvent

**Figure 2:**
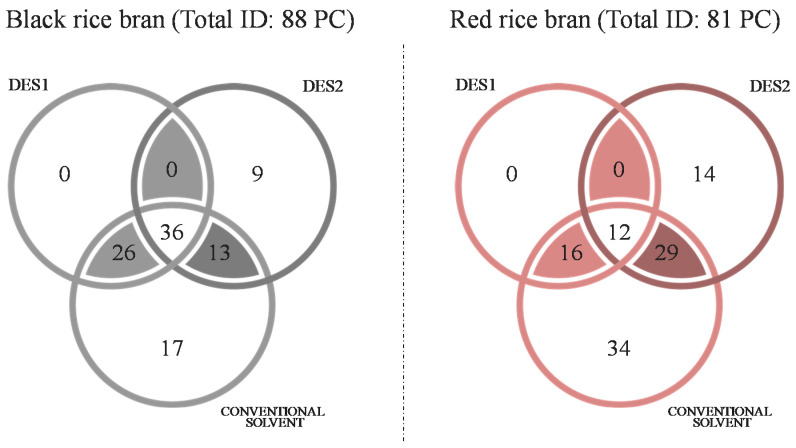
Venn diagrams of identified phenolic compounds by pigmented RB in the different extracts.

To better visualize common and unique PCs within the different extracts, PCs were displayed under the form of a Venn diagram (Figure 2). When looking at PCs in common in the three types of extracts either for black RB or for red RB, 36 common PCs out of 88 were identified in black RB versus 12 only out of 81 in red RB. This limited common pool of PCs underlines the specificity of extraction of each type of extract.

Focusing on unique compounds this time, the Venn diagram indicated some pools of unique compounds for conventional and DES2 (choline chloride/lactic acid, 1:10) extracts only. Unique PCs extracted with conventional solvents and identified by MS will not be discussed further since they have already been evidenced in the literature [5,11,32]. Considering DES2 solvent, it allowed the extraction of additional and unique compounds as follows, nine unique PCs were obtained from black RB and 14 from red RB. The acidity of the DES2 is probably effective in triggering bound phenolic acid hydrolysis and their release in the extract, as already pointed out by Ruesgas-Ramón et al. [23]. Loypimai, et al. [34] compared different solvents and showed that acidified solvents (with added HCl) led to better yields of PCs extraction. In our study, we also observe that DES2 is able to extract glycosylated or ester cyclic molecules. In comparison, no unique PC was identified in DES1 extracts which had a pool of PCs in common with conventional extracts (respectively 26 for the Black RB and 16 for red RB).

If we try to identify these unique PCs in DES2, some of these PCs had already been described in the literature for their bioactivities. For instance, dihydroresveratrol (*m/z* 229.0871 [M–H]^−^), rosmarinic acid (*m/z* 359.0775 [M–H]^−^), and the paeonol (*m/z* 165.0545 [M–H]^−^) are known to be biomarkers of inflammation pathways, protecting the nervous and cardiovascular system [35,36], as well as inducer of apoptosis and proliferative inhibitor in liver and kidney [37]. Syringaresinol (*m/z* 417.1560 [M–H]^−^) is a lignin compound that also plays a key role in inhibiting the proliferation of cancer cells [38], but it can also play an important biotechnological role. 

Indeed, Janvier, et al. [39] has shown that it can be an interesting substitute to the synthetic bisphenol A (BPA) compound in the polymer production. Eriodictyol (*m/z* 287.0567 [M–H]^−^), being one of the precursors of anthocyanins [40], is a flavonoid of a great importance. It is one of the main PCs present in citrus fruits, and exhibited antioxidant, antimicrobial, anti-inflammatory and antidiabetic activity [41,42]. It is used to mask bitter taste in beverages and in pharmaceutical industries [43]. Therefore, we have evidenced that DES2 is a good medium to extract specific RB PCs known for their biological activities. However, this specificity will very likely be dependent on the matrix [23]. 

To conclude the comparison of extraction potentials between conventional and DES solvents, although the conventional solvent extracted a higher number of PCs compared to DES, the possibility to extract different and unique PCs in DES2 offers an interesting alternative for both, the biomass characterization and the valorization of biomass-derived bioactive molecules.

### 2.3. Multivariate Analysis from the Measured Relative Abundance of Phenolic Compounds

The application of the multiplexed UPLC-MS^E^ method has enabled to quantify the relative abundance of identified compounds from the total ion counting. In Table 1, the relative abundance was summarized by color considering the percentage of each identified compound calculated from the total relative abundance (abundance higher than 75%, between 75% and 50% and lower than 50%). 

Two isomers of dihydroxybenzoic acids were the most abundant and prevalent compounds in this study. According to the literature, one may suppose that these isomers were protocatechuic and gentisic acids [10,44]. Indeed, Zarei, Luna, Leach, McClung, Vilchez, Koita and Ryan [5] evidenced that protocatechuic acid was the most abundant PCs of the pigmented RB varieties and was even present in larger quantities in black rice.

**Figure 3:**
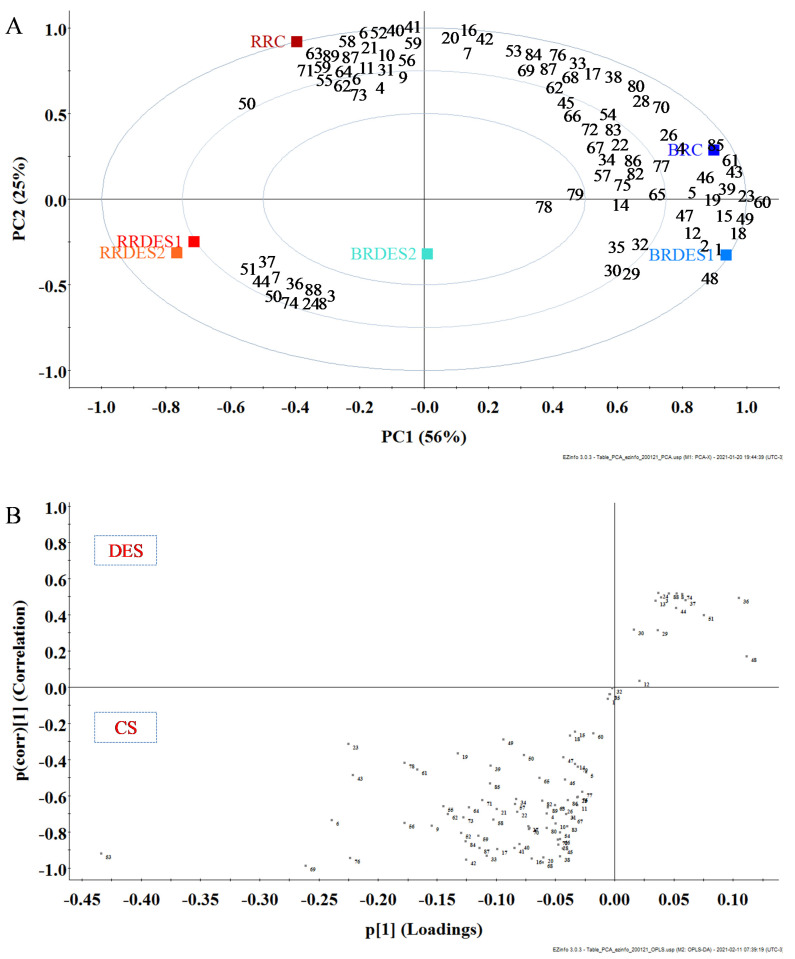
(**A**) Principal components analysis of PCs of pigmented RB extracted with conventional and deep eutectic solvents (DES) methods; (**B**) S-plots from Orthogonal Partial Least Squares Discriminant Analysis (OPLS-DA) modeling of conventional solvent (CS) extracts versus DES extracts. The loadings are represented by the respective numbers to each putative phenolic compound described in Table 2. Abbreviations: red rice conventional (RRC), red rice DES1 (RRDES1), red rice DES2 (RRDES2), black rice conventional (BRC), black rice DES1 (BRDES1), black rice DES2 (BRDES2).

To analyze the interrelations between the samples, the data were submitted to multivariate analysis by principal components analysis (Figure 3). The relative ion abundance of each putative identified PC was considered as a variable and the score of each sample was calculated (Figure 3A). The principal component 1 (PC1) accounted for 56% and the PC2 for 25% of the total variation in the dataset. It is possible to distinguish black and red RB and to highlight the differences in the PCs profile between the extraction conditions. Indeed, the red RB extraction profiles obtained with the two DES stayed close when projected on PC1 and PC2, while PC1 axis helped separating the black RB from the red RB extracts. It was marked by a high concentration in flavonoids or phenolic acids derived from hydroxybenzoic acids and chlorogenic acids. Conversely, extracts with a low score on PC1 gathered the RB extracts and were marked with lignans and phenolic acids derived from hydroxycinnamic and hydroxyphenylacetic acids. PC2 resumed less variability but separated conventional extracts, on the superior quadrant presenting high scores, from DES extracts (inferior quadrant). 

The covariance p(*1*) and correlation p(corr)(*1*) loadings from a two-class Orthogonal Partial Least Squares Discriminant Analysis (OPLS-DA) model (conventional solvent vs. DES) are displayed in a S-plot format (Figure 3B), where the variables (squares) are represented by the putative PCs. The upper right quadrant of the S-plot showed the PCs, which were elevated in DES extracts, while the lower left quadrant presented the PCs which were elevated in conventional solvent extracts. The measured intensities and factor of changes were based on the average of the measured values for each PCs in the group. To ease the reading of such an S-plot, we must specify: the further away from the *x*-axis the compound is, the greater the contribution to the variation between the groups, while the further away from the *y*-axis, the greater the reliability of the analytical result, thus the significance. The two most important PCs that explained differences between DES and conventional extracts were the coumaric acid (−0.44; −0.95) and the syringaldehyde (−0.26; −1).

### 2.4. Further Characterization of Extracts

#### 2.4.1. Quantitative Examination of the Typical Phenolic Acids Found in Cereals

**Figure 4:**
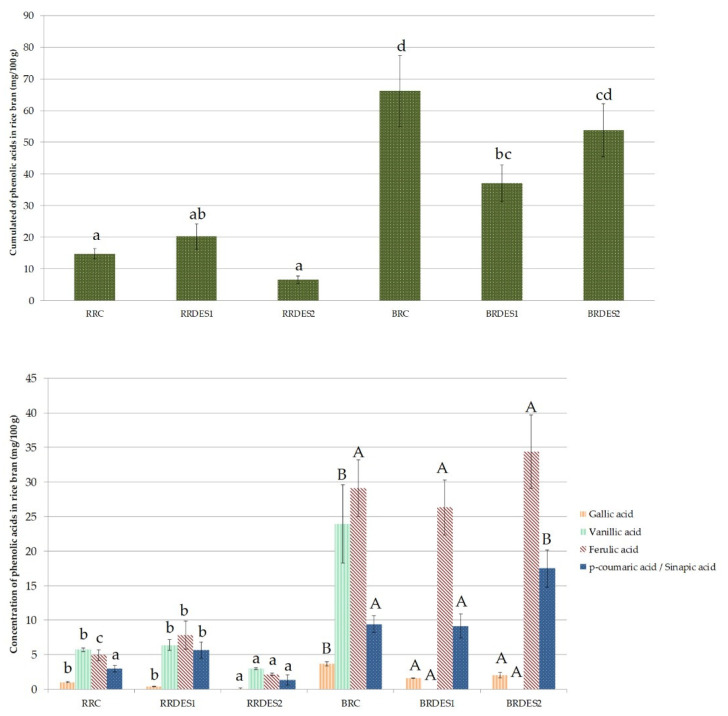
Concentration (mg/100 g) of RB extracts in five typical phenolic acids found in cereals. (Figure on the top) Cumulated concentration of the five phenolic acids quantified in this study. Different lowercase letters between extracts indicate significant differences in cumulated concentration of the amount of the five phenolic acids (*p* < 0.05). An extract with "ab" means that this concentration doesn’t have a significant statistical difference with the compared concentrations which have the same letter (a and b). (Figure at the bottom) Quantification of gallic, vanillic, ferulic and p-coumaric/sinapic acids in RB extracts. Different lowercase letters indicate significant differences between red RB extracts for a given phenolic acid. Different uppercase letters: indicate significant differences between black RB extracts for a given phenolic acid (*p* < 0.05). Abbreviations: red rice conventional (RRC), red rice DES1 (RRDES1), red rice DES2 (RRDES2), black rice conventional (BRC), black rice DES1 (BRDES1), black rice DES2 (BRDES2).

The quantification of five typical phenolic acids (gallic, vanillic, *p*-coumaric, sinapic and ferulic acids) in the different extracts of black and red RB was carried out and is displayed in Figure 4. These phenolic acids were selected because they had already been found in the pigmented RB [9,11,45,46]. The quantification profile of those phenolic acids was different and dependent of the extraction conditions. In this work, conventional solvent and DES1 resulted in extracts with higher content of these five phenolic acids in comparison with DES2. This study points out that some DES solvents can have high extraction capacity leading to broad profile of extracted PCs and can thus be good substitutes for conventional solvents while other DES formulations can lead to narrower and more specific PC extraction profiles.

Moreover, such quantification of phenolic acids allowed strongly differentiating red and black RB extracts (Figure 4). Indeed, whatever the solvent used for extraction, red RB extracts were less concentrated in phenolic acids than the black RB extracts. These results were coherent with literature which has already demonstrated that among colored rice, black rice outer layers contains more phenolics than red rice [46]. Black RB extracts contained sixfold more phenolic acids than red RB, with a total ranging between 37–66 mg/100 g in black RB extracts vs. 6–20 mg/100 g in red RB extracts. These contents were in agreement with other authors in literature [45]. Among these compounds (Figure 4), the ferulic acid was the most abundant in most cases (26–34 mg/100 g of black RB vs. 2–8 mg/100 g of red RB). Ferulic acid was indeed reported to be the main phenolic acid in cereals, especially in the bound fraction [12]. The second most concentrated phenolic acid quantified was vanillic acid in red RB, and *p*-coumaric/sinapic acid in black RB. These concentrations differed depending on the combination of solvent type (conventional solvent vs. DES1 vs. DES2) and of the type of matrix. Our results showed lower levels in vanillic acid in red RB compared with black RB in conventional extracts. Such result contrasted from the ones of Shao, Xu, Sun, Bao and Beta [44] who reported that vanillic acid was found only in black rice. Gallic acid was the least concentrated phenolic acid among the five quantified compounds, being more concentrated in black RB than in red RB, in agreement with Shao, Xu, Sun, Bao and Beta [11].

#### 2.4.2. Scavenging Ability of Rice Bran Extracts Assessed by the DPPH Radical Assay

DPPH assay was conducted to assess the reducing power of the RB extracts by different solvents. The EC_50_ was expressed as equivalent of RB extract in mg/mL.

EC_50_ of 0.26 and 0.18 mg/mL were obtained for black RB DES1 and DES2 extracts respectively (*p* < 0.01). In comparison, higher EC_50_ of 0.46 and 0.36 mg/mL were obtained for red RB DES1 and DES2 extracts, respectively (*p* < 0.01).

Two conclusions can be drawn from these results:

(1) Whatever the DES, black RB extracts have higher capacity to reduce the DPPH radical, i.e., lower EC_50_, in comparison with red RB extracts (*p* < 0.01). Such higher reducing capacity is coherent with black RB highest load in PCs [47]. We must precise that at concentrations of black RB extracts of 0.50 mg/mL, total reduction of DPPH was already reached and very likely explained by the high PC content of the extract. A review conducted by Goufo and Trindade [48] has similarly shown that the EC_50_ of pigmented RB was 16 times lower than of nonpigmented RB extracts. Other studies have shown that the highest antioxidant capacity of pigmented rice compared to nonpigmented rice [16] was due to the presence of proanthocyanidins and anthocyanins. Such compounds would be abundant in red rice [49] but even more in black rice [50,51].

(2) Whatever the type of RB, DES1 RB extracts showed the best results in comparison to DES2 RB extracts. Control experiments were also conducted using DES only to determine their individual contribution to DPPH reduction. These control experiments pointed out that DES solvent on their own were responsible for 16% and 37%, DES1 and DES2 respectively, of the reducing capacity in these extracts.

## 3. Materials and Methods

### 3.1. Chemicals

Choline chloride (≥99%), lactic acid (∼90%), 1.2-propanediol (≥99%), ethanol (reagent), methanol (CHROMASOLV^®^  ≥ 99.9%), water (CHROMASOLV^®^ Plus grade HPLC), 2,2,1-diphenyl-1-picrylhydrazyl (DPPH), 6-hydroxy-2,5,7,8-tetramethylchroman-2-carboxylic acid (Trolox) gallic, ferulic, *p*-coumaric, sinapic and vanillic acids were obtained from Sigma-Aldrich (St. Louis, MO, USA).

### 3.2. Pigmented Rice Brans

Red (*cv. TamTam*) and black (*cv*. *Artemide*) rice grains cultivated in Camargue were provided by the “Centre Français du Riz” (Arles, France). Grains (5 kg) were subject to dry abrasion using a DMS 500 huller (Electra, Poudenas, France) to obtain a fine bran fraction corresponding to between 4–7% of the grain mass. Acronyms were used to identify the analyzed rice bran extracts throughout the document: red rice conventional (RRC), red rice DES1 (RRDES1), red rice DES2 (RRDES2), black rice conventional (BRC), black rice DES1 (BRDES1), black rice DES2 (BRDES2).

### 3.3. Preparation of Deep Eutectic Solvents

Two choline-chloride based DES in combination with lactic acid or 1.2-propanediol were selected based on GRAS components and according to the previous study from Ruesgas-Ramón et al., 2017. DES were prepared by heating (60 °C), agitating (400 rpm) (IKA KS 4000 I control, Staufen, Germany) and mixing the components at the corresponding mole ratios (Table 1) in a closed bottle for 45 min until a clear liquid is formed [23]. The water content (Coulometer Karl Fisher GRS 2000, KF TITRATOR, Bioblock scientific, France), water activity (a_w_) (Aqualab, Decagon Devices Inc., Pullman, WA, USA) and pH (EcoScan pH 5 Palmtop pH-meter, Legallais, Montferrier-sur-Lez, France) were determined (Table 2).

### 3.4. Extraction of Phenolic Compounds from Rice Brans

One hundred mg of pigmented RB were extracted in triplicate with 2 mL of DES1 or DES2 or conventional solvent (ethanol/water, 60:40 *v/v*) in a closed amber glass flask in an orbital agitation (400 rpm, 40 °C, 25 min) (Cimarec Thermo Scientific Poly 15, Legallais, Montferrier-sur-Lez, France). The samples were cooled to room temperature (~20 °C) and centrifuged for 5 min at 4000 rpm (CR412 centrifuge; Jouan, Winchester, VA, USA). The supernatant was filtered with a cellulose filter (0.45 µm) (Minisart Legallais, Montferrier-sur-Lez, France), dried under nitrogen stream and then dried extracts were stored at −20 °C.

### 3.5. HPLC-DAD Characterization

The obtained extracts were analyzed in triplicate in a HPLC (LC-20AD with oven: CTO-10ASvp and detector DAD SPO-M20, Shimadzu, Noisiel, France) at 280 nm. Conventional solvent extracts were dissolved in a MeOH/water (2:1, *v/v*) and filtered on 0.45 μm cellulose filter (Minisart Legallais, Montferrier-sur-Lez, France). Those obtained by DES were diluted five times in water and then injected. The separation was carried out on a C18 column (Kinetex High purity, 5 µm, 100 A, 250 × 4.6 mm, Thermo Electron, Burlington, MA, USA) with the mobile phase A (MeOH, 0.1% acetic acid) and B (water, 0.1% acetic acid), flow rate of 1 mL/min and gradient method: 0–5 min: isocratic at 10% of B; 5–20 min: linear gradient up to 100% B; 20–30 min isocratic at 100% B; 30–35 min linear gradient up to 10% B; 35–42 min: equilibration at 10% B. Quantification of phenolic acids was performed by using a standard calibration curve made with different concentrations (0.01–3 mg/mL) of pure ferulic, *p*-coumaric, sinapic, vanillic and gallic acids.

### 3.6. Metabolomic Analysis and Data Processing

Analyses were performed by UPLC Acquity (Waters, Milford, CT, USA) coupled to the Xevo G2-S Q-Tof (Waters, Manchester, UK) equipped with an electrospray ionization source and acquired using a multiplexed MS/MS acquisition with alternating low and high energy acquisition (MS^E^). Data were processed by Progenesis QI (NonLinear Dynamics, Waters) with the PubChem and Phenol Explorer online database according to Santos, et al. [52].

### 3.7. DPPH Assessment of the Reducing Power of Extracts

The DPPH scavenging antioxidant activity was estimated according to the traditional method [29] but adapted to microplate assay (TECAN, Infinite M1000 PRO, Gröedig, Austria). Briefly, 20 μL of the samples and 180 μL of a methanolic solution of DPPH (final concentration in well 150 µM) were added on microplates (ThermoFischer, Courtaboeuf, France) and the absorbance was immediately read at 515 nm, every 5 min for the first 20 min, and then every 20 min for 1 h. Blanks with DES or ethanol/water 60:40 *v/v* were carried out to evaluate and to subtract the reducing activity of solvents. EC_50_ corresponds to the concentration of rice bran extracts (mg/mL) able to reduce 50% of the initial DPPH. Assay was performed at 37 °C in triplicate for each sample.

### 3.8. Statistics Analysis

EZInfo v. 3.0.3 (Umetrics, Sweden) was used for the analysis of metabolomics multivariate data. Principal Component Analysis (PCA) using pareto-scaling [53] and S-plot by Orthogonal Partial Least Squares Discriminant Analysis (OPLS-DA) were generated from UPLC-MS^E^ data. Matrices of data gathered all compounds’ abundances for each type of solvent and the type of pigmented rice. The HPLC and DPPH data were submitted to one-way ANOVA (Tukey, *p* < 0.05) by using R statistics (v 4.0.2).

## 4. Conclusions

In the present study, complementary techniques were applied to characterize pigmented RB extracts obtained either with conventional or DES extraction procedures. Use of modern analytical tools allowed the quantification of the most typical phenolic acids in rice as well as the description of the PC present in the different extracts. This approach underlined the high extraction capacity of DES, and most importantly, the extraction specificity of acidic DES for certain PCs. Whatever the solvent (conventional or DES), black RB remained the most enriched in PCs, and the source of RB with the highest potential for applications in human nutrition. To the best of our knowledge, this work was the first omics approach to characterize PCs in the pigmented RB DES extracts. In addition, a DPPH assay revealed that these pigmented RB extracts in DES presented an interesting reducing power, which opens the way to conceive new-pigmented RB formulations with these innovative liquid mixtures. This approach of extraction and omic characterization of extracts should be repeated on other pigmented rice varieties including purple, black-purple, orange or brown variants, known for their high load in flavonoids, anthocyanin and proanthocyanidin [14]. Therefore, more extensive works are currently under investigation to develop dietary or pharmaceutical formulations to improve the health benefits associated with the presence of these bioactive compounds.

## Figures and Tables

**Table 1:** Putative identification of phenolic compounds (PCs) in pigmented rice bran (RB) extracts by UPLC-MS^E^.

	Putative Compound	[M – H]^−^	RT (min)	Molecular Formula	Score	FS	Fragments /Intensity	ME	IS	Black Rice Bran			Red Rice Bran		
										CS	DES1	DES2	CS	DES1	DES2
1	Gallic acid *	169.0131	1.26	C_7_H_6_O_5_	37.3	0	Nd	−6.89	94.40						
2	Dihydroxybenzoic acid isomer I *	153.0181	1.41	C_7_H_6_O_4_	37.1	0	Nd	−8.25	94.68						
3	4-Hydroxymandelic acid/Vanillic acid isomer I	167.0334	1.46	C_8_H_8_O_4_	36.1	0	Nd	−9.59	90.85						
4	Dihydroxybenzoic acid isomer II	153.0180	1.48	C_7_H_6_O_4_	37.1	0	Nd	−8.46	94.67						
5	Caffeoylquinic acid isomer I	353.0863	1.54	C_16_H_18_O_9_	35.5	0	Nd	−4.19	82.23						
6	4-Hydroxymandelic acid/Vanillic acid isomer II	167.0337	1.64	C_8_H_8_O_4_	37.8	0	Nd	−7.85	97.57						
7	Dihydroxybenzoic acid isomer III	153.0179	1.64	C_7_H_6_O_4_	36.7	0	Nd	−9.25	93.70						
8	Apigenin 7-O-glucoside	419.1351	1.73	C_21_H_24_O_9_	36.2	0	Nd	0.77	81.93						
9	Isorhamnetin/Rhamnetin/Nepetin	315.0498	1.74	C_16_H_12_O_7_	37.7	0	Nd	−3.95	93.23						
10	Irilone	297.0392	1.75	C_16_H_10_O_6_	35.6	0	Nd	−4.20	82.89						
11	Dihydro-p-coumaric acid/Methoxyphenylacetic acid	165.0545	1.81	C_9_H_10_O_3_	36.3	0	Nd	−7.41	90.07						
12	Dihydroxybenzoic acid isomer IV	153.0180	1.85	C_7_H_6_O_4_	38	0	Nd	−8.46	99.40						
13	Eriodictyol isomer I	287.0567	1.90	C_15_H_12_O_6_	38.6	11.7	165.0543 (19%)	2.05	83.77						
14	Esculetin	177.0180	1.91	C_9_H_6_O_4_	37.4	0	Nd	−7.54	95.53						
15	Quercetin 3-O-glucoside isomer I	463.0870	1.94	C_21_H_20_O_12_	37.4	0	Nd	−2.69	90.16						
16	Syringic acid/Gallic acid ethyl ester/3,4-Dihydroxyphenyllactic acid	197.0444	2.09	C_9_H_10_O_5_	36.5	0	Nd	−6.00	89.58						
17	Homovanillic acid/Dihydrocaffeic acid	181.0494	2.13	C_9_H_10_O_4_	37.6	0	Nd	−6.85	95.91						
18	Myricetin	317.0290	2.16	C_15_H_10_O_8_	38.9	5.13	124.0146 (17%), 123.0076 (11%)	−4.04	94.33						
19	Coumaroylquinic acid isomer I	337.0916	2.27	C_16_H_18_O_8_	43.2	20.7	119.0488 (100%), 191.0549 (5%), 20111.0434 (2%)	−3.70	99.55						
20	4-Hydroxymandelic acid/Vanillic acid isomer III	167.0335	2.29	C_8_H_8_O_4_	36.2	0	Nd	−9.01	90.85						
21	Cyanidin 3-*O*-beta-D-sambubioside	580.1488	2.38	C_26_H_29_O_15_+	36.2	0.631	115.0401 (2%)	9.41	90.56						
22	Methylgallic acid	183.0289	2.42	C_8_H_8_O_5_	38.6	0	Nd	−5.28	98.86						
23	Feruloylquinic acid isomer I	367.1023	2.45	C_17_H_20_O_9_	57.3	91	134.0359 (100%), 193.0494 (29%), 200.0442 (25%), 117.0333 (14%), 123.0436 (7%), 155.0335 (2%)	−3.18	99.18						
24	Dihydroresveratrol	229.0878	2.54	C_14_H_14_O_3_	36.2	0	Nd	3.43	85.26						
25	Scopoletin	191.0332	2.54	C_10_H_8_O_4_	37	0	Nd	−9.04	95.14						
26	4-Hydroxymandelic acid/Vanillic acid isomer IV	167.0337	2.56	C_8_H_8_O_4_	36.5	0	Nd	−7.53	90.85						
27	4′-O-Methylepigallocatechin	319.0809	2.58	C_16_H_16_O_7_	35.5	0	Nd	−4.54	82.64						
28	Feruloyl glucose	355.1016	2.58	C_16_H_20_O_9_	40.7	15.9	177.0545 (41%)	−5.08	93.62						
29	Bergapten/Xanthoxin	215.0335	2.60	C_12_H_8_O_4_	44.9	36.8	191.0333 (100%)	−6.78	95.46						
30	Psoralen	185.0233	2.60	C_11_H_6_O_3_	36.2	0	Nd	−6.24	88.21						
**31**	(+)-Catechin *	289.0705	2.67	C_15_H_14_O_6_	35.7	0	Nd	−4.38	83.74						
32	Kaempferide	298.0465	2.69	C_16_H_11_O_6_-	39.4	20.9	175.0388 (76%), 134.0360 (27%), 193.0127 (13%), 117.0330 (1%)	−6.01	82.88						
**33**	Caffeic acid *	179.0335	2.72	C_9_H_8_O_4_	38	0	Nd	−7.97	98.69						
34	Trihydroxyisoflavone	269.0443	2.73	C_15_H_10_O_5_	38.6	0	Nd	−4.63	98.27						
35	Isorhamnetin 3-O-glucoside/Isorhamnetin 3-O-galactoside	477.1021	2.75	C_22_H_22_O_12_	40.8	16.2	429.0818 (1%), 59.0113 (1%)	−3.68	92.19						
36	Hydroxymatairesinol isomer I	373.1303	2.77	C_20_H_22_O_7_	43.2	28.9	205.0494 (100%),223.0601 (62%), 179.0700 (12%), 221.0805 (6%), 181.0491 (4%), 193.0854 (1%), 105.0331 (1%)	2.67	90.02						
37	Syringaresinol isomer I	417.1560	2.80	C_22_H_26_O_8_	37.7	0	Nd	1.17	89.93						
38	Dicaffeoylquinic acid	515.1221	2.83	C_25_H_24_O_12_	38.4	5.72	307.0909 (3%)	4.94	92.15						
39	Coumaroylquinic acid isomer II	337.0917	2.84	C_16_H_18_O_8_	45.2	32.1	245.0803 (50%), 119.0486 (15%), 93.0327 (13%), 243.0651 (11%)	−3.56	98.01						
**40**	**(-)-Epicatechin**	289.0700	2.84	C_15_H_14_O_6_	46.9	48.8	257.0438 (100%), 243.0651 (11%)	−6.23	92.53						
41	4-Hydroxymandelic acid/Vanillic acid isomer V	167.0337	2.90	C_8_H_8_O_4_	51.6	75.8	151.0385 (100%), 123.0437 (14%), 135.0435 (4%), 105.0332 (1%)	−7.79	90.85						
42	3,4-Dihydroxyphenyllactic acid	197.0441	2.98	C_9_H_10_O_5_	44.2	34.6	134.0357 (90%)	−7.18	94.67						
43	Feruloylquinic acid isomer II	367.1023	2.99	C_17_H_20_O_9_	42.7	17.5	134.0357 (100%), 173.0443 (76%), 191.0546 (31%), 117.0330 (10%), 111.0436 (10%), 155.0332 (7%), 75.0065 (5%)	−3.03	99.41						
44	Rosmarinic acid	359.0775	3.02	C_18_H_16_O_8_	35.9	0	Nd	0.85	80.69						
45	Quercetin 3-O-rutinoside/Kaempferol 3-O-sophoroside/Quercetin 3-O-rhamnosyl-galactoside/Kaempferol 3,7-O-diglucoside isomer I	609.1446	3.21	C_27_H_30_O_16_	38	0	Nd	−2.44	92.86						
46	Tetrahydroxyisoflavone isomer I	285.0391	3.27	C_15_H_10_O_6_	47.1	52.9	151.0386 (100%)	−4.70	88.27						
47	Eriodictyol 7-O-glucoside	449.1075	3.30	C_21_H_22_O_11_	45.7	34.3	103.0386 (100%), 181.0498 (22%), 122.0359 (16%), 311.0760 (10%), 99.0075 (7%)	−3.24	97.82						
48	Dihydroxybenzoic acid isomer V	153.0181	3.31	C_7_H_6_O_4_	38	0	Nd	−8.22	99.29						
49	Quercetin 3-O-rutinoside/Kaempferol 3-O-sophoroside/Quercetin 3-O-rhamnosyl-galactoside/Kaempferol 3,7-O-diglucoside isomer II	609.1454	3.38	C_27_H_30_O_16_	42.7	16.9	300.0264 (8%)	−1.16	98.00						
50	Luteolin 7-O-rutinoside/Kaempferol 3-O-rutinoside/Chrysoeriol 7-O-apiosyl-glucoside	593.1495	3.39	C_27_H_30_O_15_	37.1	1.66	103.0387 (7%), 175.0600 (2%)	−2.81	87.33						
51	Didymin/Poncirin	593.1884	3.40	C_28_H_34_O_14_	36.7	2.47	103.0387 (100%), 175.0600 (23%)	1.41	82.63						
52	Salvianolic acid D	237.0395	3.44	C_11_H_10_O_6_	38.2	0	Nd	−3.88	95.39						
**53**	*p*-coumaric acid *	163.0389	3.47	C_9_H_8_O_3_	40.8	12.3	163.0388 (28%), 119.0488 (21%)	−6.97	99.39						
54	Phloridzin	435.1277	3.48	C_21_H_24_O_10_	37.2	7.68	103.0387 (100%)	−4.47	83.45						
55	Schisandrin B	399.1835	3.51	C_23_H_28_O_6_	37.3	0	Nd	5.39	92.86						
56	Tectoridin	461.1080	3.54	C_22_H_22_O_11_	41.8	16.4	341.0654 (4%), 146.0341 (1%)	−2.00	95.23						
57	Glycitin	445.1129	3.59	C_22_H_22_O_10_	44.3	29.7	326.0777 (29%), 283.0593 (22%), 379.0769 (16%)	−2.51	94.59						
58	Isorhamnetin 3-O-rutinoside	461.1067	3.63	C_22_H_22_O_11_	50.6	69.8	324.0255 (100%), 279.0288 (29%), 99.0070 (7%), 73.0274 (5%)	−4.82	88.73						
**59**	Ferulic acid *	**193.0495**	**3.64**	C_10_H_10_O_4_	40	10.6	137.0590 (13%)	−5.68	96.05						
60	Paeoniflorin	479.1549	3.66	C_23_H_28_O_11_	38.1	0	Nd	−2.09	92.84						
61	Tetrahydroxyisoflavone isomer II	285.0392	3.73	C_15_H_10_O_6_	39.8	5.06	117.0331 (9%), 105.0330 (4%), 132.0206 (3%)	−4.38	99.00						
62	Violanone	315.0860	3.75	C_17_H_16_O_6_	43.2	23.3	165.0543 (32%)	−4.45	98.07						
63	3,7-Dimethylquercetin/Jaceosidin/Tricin isomer I	329.0654	3.80	C_17_H_14_O_7_	37.4	0	Nd	−3.97	91.62						
64	Diosmin	607.1656	3.81	C_28_H_32_O_15_	38.6	1.83	89.0229 (100%)	−2.04	93.78						
65	Tetrahydroxyisoflavone isomer III	285.0393	3.88	C_15_H_10_O_6_	38.4	0.795	123.0074 (1%)	−4.03	95.99						
66	Schisantherin A	535.2012	3.90	C_30_H_32_O_9_	34.9	0.218	191.0701 (1%)	7.14	82.26						
67	Gardenin B	357.0968	3.91	C_19_H_18_O_7_	47.4	51.3	209.0445 (100%), 315.0861 (45%), 239.0552 (32%), 327.0860 (22%), 345.0952 (17%), 251.0550 (13%), 177.0547 (14%), 181.0491 (12%)	−3.24	89.72						
68	Hesperidin	609.1820	3.93	C_28_H_34_O_15_	36	0.274	161.0596 (4%)	−0.78	80.56						
69	Syringaldehyde	181.0495	4.29	C_9_H_10_O_4_	38.4	0	Nd	−6.18	99.23						
70	Eriodictyol isomer II	287.0549	4.34	C_15_H_12_O_6_	38.1	3.22	147.0071 (16%), 119.0123 (12%), 123.0075 (2%)	−4.19	92.29						
71	3,7-Dimethylquercetin/Jaceosidin/Tricin isomer II	329.0655	4.38	C_17_H_14_O_7_	38.3	5.77	122.0355 (3%), 146.0350 (3%)	−3.70	90.18						
72	Nobiletin	401.1229	4.52	C_21_H_22_O_8_	37.4	0	Nd	−3.14	90.76						
73	Hydroxymatairesinol isomer II	373.1281	4.62	C_20_H_22_O_7_	38.1	2.26	146.0343 (10%)	−3.23	91.85						
74	Syringaresinol isomer II	417.1560	4.74	C_22_H_26_O_8_	36.6	0	Nd	1.32	84.73						
75	Isorhamnetin isomer I	315.0495	4.79	C_16_H_12_O_7_	49.4	59.6	175.3038 (100%), 160.0152 (20%)	−4.75	92.69						
76	Tetrahydroxyisoflavone isomer V	285.0393	5.01	C_15_H_10_O_6_	42.7	21.6	133.0280 (100%), 132.0207 (9%), 179.0343 (4%)	−4.21	96.95						
77	3,7-Dimethylquercetin/Jaceosidin/Tricin isomer II	329.0648	5.04	C_17_H_14_O_7_	40	10.8	121.0281 (35%), 139.0387 (4%), 147.0434 (3%), 119.0121 (2%)	−5.78	96.01						
**78**	**Quercetin**	**301.0342**	**5.06**	C_15_H_10_O_7_	49.6	53.2	151.0023 (100%), 121.0181 (35%), 178.9973 (19%)	−3.80	99.43						
79	Dihydroquercetin *	303.0499	5.16	C_15_H_12_O_7_	38.2	0	Nd	−3.67	95.13						
80	Trihydroxyisoflavanone isomer II	271.0595	5.38	C_15_H_12_O_5_	37.4	0	Nd	−6.09	94.02						
81	Isorhamnetin isomer II	315.0498	5.56	C_16_H_12_O_7_	40.1	6.44	117.0334 (13%)	−3.73	98.49						
82	6-Hydroxyluteolin/Morin	301.0338	5.56	C_15_H_10_O_7_	37.5	0	Nd	−5.33	93.63						
83	Urolithin A	227.0337	5.56	C_13_H_8_O_4_	39.8	19	183.0435 (100%), 182.0360 (24%), 167.0486 (7%)	−5.42	86.04						
84	Trihydroxyisoflavanone isomer III	271.0598	5.64	C_15_H_12_O_5_	40	9.09	119.0487 (100%)	−4.98	96.67						
85	Hesperetin/Homoeriodictyol	301.0704	5.73	C_16_H_14_O_6_	42.8	21.1	193.0492 (23%), 134.0358 (19%), 164.0097 (7%), 149.0591 (6%)	−4.41	97.88						
86	Dihydroxykaempferol	317.0290	5.75	C_15_H_10_O_8_	42	23.2	271.0234 (100%), 107.0124 (11%)	−4.10	91.65						
87	Hispidulin	299.0545	5.80	C_16_H_12_O_6_	37.6	0	Nd	−5.51	94.43						
88	Paeonol	165.0545	6.71	C_9_H_10_O_3_	36.4	0	Nd	−7.14	90.07						
89	Rosmanol	345.1688	6.72	C_20_H_26_O_5_	37.4	0	Nd	−5.74	93.66						
**Number of identifications**										**# 79**	**# 49**	**# 58**	**# 67**	**# 16**	**# 43**
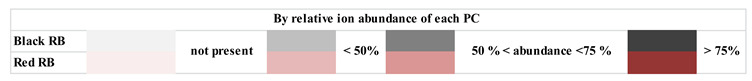															

RT: retention time; FS: fragmentation score; ME: mass error; IS: isotope similarity; Nd: Not detected. Bold: reference standards; CS: conventional solvent. * identified by Zarei, Luna, Leach, McClung, Vilchez, Koita and Ryan [5].

**Table 2:** Composition of DES and measurement results A_w_, Karl Fisher water content and pH.

Name	# 1	# 2	# 3	Molar Ratio	a_W_	Water Content	pH
DES1	Choline chloride	1.2-propanediol	Water	1:1:1	0.51 ± 0.03	7.2 ± 0.5	5.42
DES2	Choline chloride	Lactic acid	-	1:10	0.29 ± 0.00	6.3 ± 0.25	--

Abbreviations. a_W_ = water activity, **#** = component, --: negative values linked to very acidic and specific medium.

## Data Availability

No new data were created or analyzed in this study. Data sharing is no applicable to this article.
